# Establishing and reviewing podiatry service to the haemodialysis ward at Caulfield Hospital

**DOI:** 10.1186/1757-1146-4-S1-P1

**Published:** 2011-05-20

**Authors:** Ana Andric, Katie Hjorth, Gillian Shaw

**Affiliations:** 1 Caulfield Hospital, 260 Kooyong Rd Caulfield, 3162, Australia

## Background

Podiatry services for patients attending haemodialysis at Caulfield Hospital (CH) began in June 2009 due to emerging evidence indicating people with chronic kidney disease and end stage renal failure are at an increased risk of developing lower limb complications such as ulceration and amputation. Risk factors become more frequent with progression of the disease, some research also suggests 95% of people with diabetes who receive haemodialysis treatment have at least one risk factor for foot ulceration.

## Methods

Preliminary patient surveys were undertaken to establish patient interest in receiving podiatry treatment. Prior to receiving treatment all patients underwent an initial podiatry assessment establishing their baseline vascular and neurological status and other information to help determine their level of risk. Following a 12 month pilot further process evaluation data was collected.

## Results

An overwhelmingly positive response was received from patients and haemodialysis nursing staff in requesting access to podiatry services. At the time of the initial survey only 43% of haemodialysis patients were accessing podiatry. A haemodialysis podiatry ward round has been established on alternating days and weeks with an 8 weekly review period to ensure easy access for all patients. Treatment is provided at no cost to the patients and takes place during haemodialysis treatment hours; making it more accessible and convenient for patients. Additional funding was not obtained for this service and was redistributed from existing funding streams. A 12 month service review was conducted in July 2010 surveying podiatry staff, haemodialysis nursing staff and patients. The feedback from all parties was positive and encouraging and it was decided the haemodialysis ward round would continue with some minor changes. The survey revealed the percentage of people accessing podiatry services had increased to 73% (from 43%) within one year; 91% of these patients reported receiving podiatry treatment whilst attending haemodialysis at CH. Most recently funding has been procured to implement a limited podiatry service at Sandringham Hospital. The service will include access for patients receiving haemodialysis and will utilise the model previously piloted at CH.

## Conclusions

The future aim is to obtain funding for podiatry services for haemodialysis patients across all Alfred Health sites and encourage more patients to access the service by ensuring relevant information and education is provided. Ethics approval has been sought to further investigate the effects of haemodialysis on the foot. The scope of this would include increasing awareness amongst the greater community of foot complications in haemodialysis patients and assist in establishing better access to podiatry services for patients undergoing haemodialysis.

